# Innovative Covering Liquids Stabilising Water–Fat Leachate from Fresh Mozzarella Cheese Used as Pizza Topping

**DOI:** 10.3390/foods13040581

**Published:** 2024-02-14

**Authors:** Jakub Biegalski, Dorota Cais-Sokolińska

**Affiliations:** Department of Dairy and Process Engineering, Faculty of Food Science and Nutrition, Poznań University of Life Sciences, ul. Wojska Polskiego 31/33, 60-624 Poznań, Poland; cais@up.poznan.pl

**Keywords:** mozzarella cheese, covering liquid, pizza baking, water diffusion, leachate

## Abstract

This study analyses the possibility of changing the composition of the covering liquid in which mozzarella cheese is stored. The characterisation of mozzarella cheese consumed fresh and during later culinary use as a pizza topping was carried out. Mozzarella cheese from cow’s milk and reconstituted sheep’s milk were used for this study. The cheese was stored in whey-based covering liquid to which single or double amounts of lactose and/or citric acid (*w*/*w*) were added. The results obtained during laboratory analysis showed that the addition of lactose and/or citric acid to the covering liquid significantly impacted the mass of the cheese and the changes that can occur during later culinary use. The observed changes in the cheese during storage in the covering liquid were confirmed by the characteristics of the liquid itself. The smallest mass changes were related to cheeses stored in a covering liquid with double the amount of lactose and a single amount of citric acid. This cheese also displayed positive changes in all assessed descriptors (texture, melt, and colour). The amount of leachate from the cheese was small and occurred relatively late after unpacking and quartering. Based on the results of the study, changes made to the composition of the covering liquids can positively affect the characteristics of mozzarella cheese. Additionally, this data allows for the creation of specialised mozzarella cheeses that can pique the consumer’s interest.

## 1. Introduction

Mozzarella cheese is usually packed and stored in a covering liquid in the form of water, stretching water, whey or diluted brine [1,2,3] to preserve the delicate taste, freshness and springiness [2,4]. The liquid used for mozzarella cheese packing has multiple functions. It protects the delicate structure of the cheese during distribution and stabilises its composition and properties during storage. However, this type of packaging increases perishability, resulting in traditional mozzarella cheese having a shelf life of 1 to 2 weeks [4,5]. Manufacturing procedures and storage conditions can also positively or negatively influence this time period [4]. For example, a storage temperature that is too high may shorten shelf life due to microbiological and textural changes.

Preserving liquid is very important due to chemical reasons and the possible growth of microorganisms [1]. Brine, among others, is used to regulate the salt content in mozzarella cheese, which directly impacts proper moisture control, flavour, textural properties and the overall characteristics of the produced cheese. The process of cheese brining can promote the formation of a soft, moist and fragile surface layer, which is considered a defect [6,7,8]. The composition of the covering liquid is very diverse. It is usually a mixture of brine with mono, divalent salts and/or organic acids (NaCl, CaCl_2_ and calcium lactate) [1,8,9,10]. Kindstedt et al. [11] indicated that the phenomenon of generating a fragile surface layer of mozzarella cheese may be related to the calcium content. Calcium is known to play a very important role in structure formation [12]. Hence, CaCl_2_ is traditionally added to the brine, which minimises the risk of soft rind [7]. The liquid used for mozzarella cheese storage stimulates the quality and stability of the cheese during storage, which is important for further culinary use as well.

Mozzarella cheese is a popular dairy product used, among others, in dishes such as pizza, calzone and related foods. This product is characterised by appropriate functionalities both in pre- and postmelted forms [13,14,15,16]. One of the most important features of mozzarella cheese after baking is its complete melting, free oil formation, shred fusion, and desired browning [17,18]. Furthermore, many studies have investigated the extent of blistering during the baking process [16,19,20].

The methods of storing mozzarella cheese are constantly being improved. There is currently a trend towards vacuum packaging of fresh mozzarella cheese, which, however, changes its sensory characteristics. This indicates the need to improve the storage of mozzarella cheese in covering liquid. To the best of our knowledge, there is no information on the use of lactose content and acidity of the covering liquid to create unique conditions that may affect the reduction in water–fat leachate from the cheese mass. Moreover, there are no studies that examined the possibility of using such covering liquids to store fresh pasta filata cheese made from fresh cow’s milk and sheep’s milk powder, which may be a novel food product that meets the needs of the modern consumer.

In this study, we analysed the possibility of using lactose and citric acid to modify the composition of the covering liquid in which mozzarella cheese is stored. The modification was intended to reduce the amount of leachate from mozzarella cheese and improve its characteristics when later used as a pizza topping.

## 2. Materials and Methods

### 2.1. Cheese Making Protocols

The cheese was produced using fresh, raw cow’s (CS) milk and sheep’s milk powder after reconstitution (SR) in a ratio of 70:30 (CS:SR). A detailed description of the milk used for the production of the cheese was described by Biegalski and Cais-Sokolińska [21]. The procedure of making pasta filata cheese was described by Biegalski et al. [22]. The produced cheeses were shaped into spheres (220 g, Ø = 7 cm).

### 2.2. Covering Liquid Preparation

For the preparation of the covering liquid, whey remaining after the production of cheese was used (whey composition in g/kg: fat 2.0, total protein 4.9, lactose 40.5). The whey was filtered using a strainer (square perforation 0.1 × 0.1 mm) to remove larger remaining particles. The covering liquid was then prepared using ingredients and amounts (*w*/*w*) presented in Table 1. A total of 10 L of whey was used in each production batch. NaCl (0.4%) and CaCl_2_ (6.7 g × L^−1^) were also added to each mixture in the same amount. Pasteurisation of the liquid was carried out at 80 °C for 10 min.

### 2.3. Experimental Design

After the specified storage period, an analysis of the physicochemical properties of the covering liquids and mass changes in the cheese samples was carried out. A reference variant of the liquid and the cheese stored were selected and subjected to further analysis. The test specimens were taken from different production batches (*n* = 6). The cheeses were packed in covering liquid and stored at 3 °C for 5 days. PA/PE bags with a thickness of 0.08 mm were used. The cheese was prepared on a pilot plant scale, and each batch was analysed twice.

### 2.4. Water Activity, Acidity and Freezing Point

The water activity was measured using an AquaLab Series 4TE instrument (Decagon Devices Inc., Pullman, WA, USA). The pH was measured using a CP–402 pH-meter (Elmetron, Zabrze, Poland). The freezing point of the studied covering liquids was determined according to the ISO 5764 [23] standard method using an Advanced^®^ Model 4D3 (Advanced Instruments Inc., Norwood, MA, USA).

### 2.5. Electrical Properties

Conductivity in mS/cm was measured using a CP-505 conductometer (Elmetron, Zabrze, Poland). Salinity, as chloride content, was measured using a salinity meter CC-103 in g/dm^3^ (Elmetron, Zabrze, Poland).

### 2.6. Mass Changes during Storage in Covering Liquids

Changes in mass (g) of mozzarella cheese samples were assessed before and after 5 days of storage in the prepared covering liquids. WPA 180/C/1 laboratory scale (Radwag, Radom, Poland) was used for measurements.

### 2.7. Water–Fat Serum Release

The cheese was quartered by cutting it into 4 equal parts (internal layer surface = 153.9 cm^2^). The release of water–fat serum was observed as it’s leachate at refrigerated temperature. The liquid phase (mL) was collected using a microliter syringe model 702N (Hamilton, Reno, NV, USA) after cheese unpacking every 1 h for 24 h (according to preliminary research, it is the maximum storage time of the cheese after unpacking by the consumer). During the observation, the moment of the first signs of leachate was recorded.

### 2.8. Water Mobility

To measure water transport/mobility, an AWC-11 water activity meter (Cobrabid, Poznań, Poland) equipped with a Rotronic probe was used. During a 780-min period, the value of instantaneous water activities was recorded every 10 min. The results were presented in the form of a curve consisting of three areas: the first area was the constancy of the meter, the second area ended with the achievement of equilibrium water activity (differences in water activity were less than 0.001), and the third area was related to surface processes, i.e., evacuation of water outside the sample, a_w_ = const.

### 2.9. Pizza Baking Test

The pizza baking test was used to evaluate mozzarella cheese as a pizza topping. For this purpose, the methodology described by Rudan and Barbano [24] with modifications was used. Pizza was made by spreading 120 g of tomato sauce (GustoBello, Jeronimo Martins Polska S.A., Kostrzyn, Poland) on a round 25 cm pizza crust (GustoBello, Jeronimo Martins Polska S.A., Kostrzyn, Poland). Pizza crust ingredients: wheat flour, water, extra virgin olive oil, salt, yeast. Mozzarella cheese was cut into 1 cm thick slices. Slices of equal diameter Ø = 3 cm were cut from each slice and placed on the pizza crust. Each slice passed through the geometric centre of the entire mozzarella cheese ball. All pizzas were baked using the same conditions in a multifunctional electric oven Model 225929 (Hendi B.V., De Klomp, The Netherlands).

### 2.10. Descriptive Analysis of the Appearance of Cheese after Baking on Pizza

The direction of changes in the main descriptors of texture, melt and colour of mozzarella cheese stored in covering liquids with a modified composition after baking on pizza was assessed using the following markings: “0” as no change, “+” as improvement, “–” as deterioration. All changes were assessed in relation to the W control sample. The assessment was performed by a team of panellists (12; M_age_ = 34).

### 2.11. Colour Measurement

The instrumental colour measurement was based on the CIELab system described by Cais-Sokolińska et al. [25].

### 2.12. Statistical Analyses

The influence of different innovative covering liquids on the characteristics of the pasta filata cheeses was evaluated by one-way analysis of variance (ANOVA). The influence of the covering liquid composition on changes in the mass of cheese samples was assessed using the *t*-test for samples independent of the variables. Statistical analysis was carried out using TIBCO Statistica data analysis software (TIBCO Software Inc., Palo Alto, CA, USA; α = 0.05).

## 3. Results and Discussion

### 3.1. Physicochemical Properties of Covering Liquids and Mass Changes in Cheese Samples

**Figure 1:**
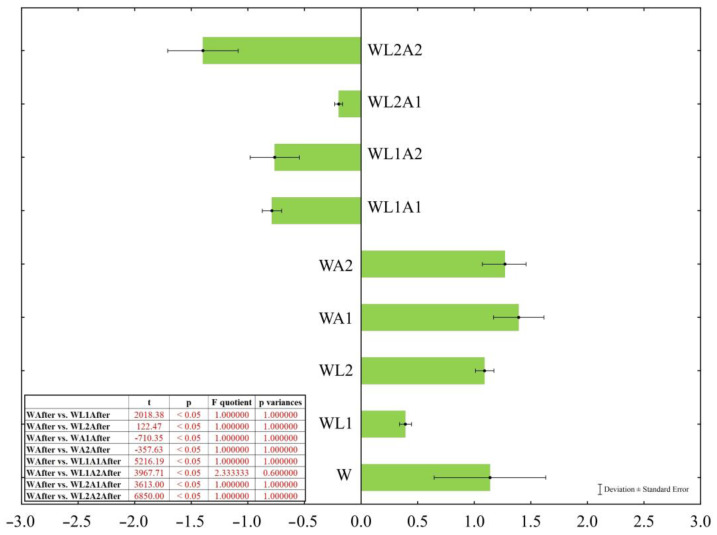
Deviation from the initial mass of the cheese samples (%) after storage in covering liquids with modified composition. The *t*-test values. Sample coding as in Table 1.

After placing the cheeses in their designated covering liquids, significant changes in the cheese’s mass and liquid properties were observed (Table 2). When the obtained values were compared with that of the control sample (W) using an independent samples test, it highlighted that statistical differences were significant (*p* < 0.05). Hence, the influence of modifying the covering liquid’s composition on the cheese mass was evident. The *t*-test values are shown in Figure 1. The mass of the cheese placed in the control liquid without additives (W) showed a 1.14 ± 0.49% increase (from 220.04 ± 0.02 g to 222.55 ± 3.68 g; *p* < 0.05). Analysis of the properties of this liquid revealed that its water activity decreased (from 0.9906 ± 0.001 to 0.9843 ± 0.002; *p* < 0.05), pH increased (from 6.521 ± 0.00 to 6.672 ± 0.02; *p* < 0.05), the freezing point decreased (from −0.6761 ± 0.00 °C to −0.6952 ± 0.00 °C; *p* < 0.05), and the salinity reduced (from 8.70 ± 0.0 g/dm^3^ to 8.40 ± 0.0 g/dm^3^; *p* < 0.05), resulting in diminished conductivity (from 35.62 ± 0.3 mS/cm to 34.45 ± 0.1 mS/cm; *p* < 0.05; *p* = 0.002).

Acidification of the covering liquid via the addition of citric acid to pH 5.89 (WA1) and 5.35 (WA2) increased the mass of the cheeses. This increase was twice as high as in the control sample, and the observed trend in changes in the liquid properties was consistent with those of the control sample. Additionally, the amount of added acid did not influence the final values of water activity, freezing point, salinity and conductivity of WA1 and WA2 covering liquids (*p* > 0.05).

There were no differences in properties between the control liquid and the liquid with the addition of lactose in the amount of 10 g/kg, both before and after storing the cheese in it (*p* > 0.05). However, the addition of lactose in a larger amount (20 g/kg) decreased the covering liquid’s pH and lowered the freezing point, as well as the salinity and conductivity (*p* < 0.05). In this case, the mass of the cheese stored in this liquid increased (*p* < 0.05). The simultaneous addition of lactose and acidification of the liquid, compared with the control sample (W), resulted in reduced pH, water activity and lower freezing temperature, as well as salinity and conductivity. However, the changes that occurred in the liquid after storing the cheese, even though they had the same direction, depended on the amount of added lactose and acid. Furthermore, the configurations of these two additives determined the changes in the mass of stored cheeses. The most significant change in cheese mass reduction was observed after storing in the WL2A2 covering liquid (*p* < 0.05), in contrast to the WL2A1 covering liquid, where the cheese almost did not change its mass.

The addition of lactose (20 g/kg) and citric acid (10 g/kg) to the WL2A1 covering liquid resulted in a significant reduction in pH to 5.82 compared to the control liquid (pH 6.52; *p* < 0.05). The freezing point also decreased significantly from −0.676 °C (W) to −0.793 °C (WL2A1). The cheese placed in WL2A1 liquid showed no significant change in its mass (*p* > 0.05; *p* = 0.424). The water activity of this liquid did not change due to the storage of cheese. However, after storage, a significant increase in pH was found (from 5.83 to 6.40; *p* < 0.05). Based on the results, due to the smallest changes in the mass of the cheeses, WL2A1 covering liquid was selected for further analysis of processes related to diffusion and water mobility.

**Figure 2:**
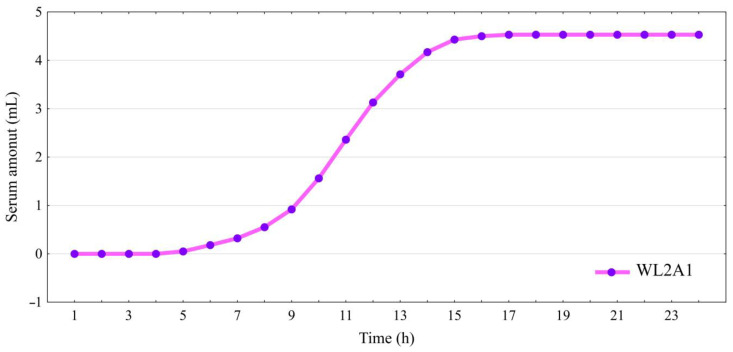
Diffusion of water–fat serum from cheese made from a mixture of cow’s milk and reconstituted sheep’s milk (70:30) stored in WL2A1 covering liquid. Sample coding as in Table 1.

When storing cheese, e.g., in salted brine, water is transferred from the cheese into the brine, which is the result of osmotic processes driven by the high salt content (>20%) in the brine [26]. When storing cheese in a covering liquid with low salt content, the osmotic effect is much smaller and water transfer occurs in the opposite direction, i.e., from the liquid to the cheese. This ultimately increases the mass of the cheese [26], which is consistent with the results obtained from the control sample (W) and samples with the addition of only lactose or acid (WL1, WL2, WA1, and WA2; Figure 1 and Figure 2). Alinovi and Mucchetti [8] proved that storing mozzarella cheese in a covering liquid in freezing conditions promoted an increase in cheese mass by up to 6.9%. This was further evidence of the relationship between the mozzarella cheese stored in the covering liquid and the covering liquid itself, which ultimately affects the weight of the cheese.

The review of the literature (e.g., [8,16,27,28]) showed that previous scientific research focused on the quality of the cheese itself. However, there is no clear information indicating how the covering liquid changes during storage and how these changes may correlate with changes occurring in the cheese, which may affect later culinary processes. This is important for the further use of the covering liquids after cheese production in accordance with the concept of a closed and antiwaste economy. Additionally, it should be noted that existing research was conducted mainly on cheese stored in traditional brine, to which NaCl was mainly added (e.g., [28,29]). This data shows that research on the quality of the covering liquids and the impact of these liquids on the quality of the cheese is necessary, especially now when the consumer expects the possibility of using the product losslessly in accordance with observed trends such as “zero waste”.

### 3.2. Water Diffusion and Mobility

**Figure 3:**
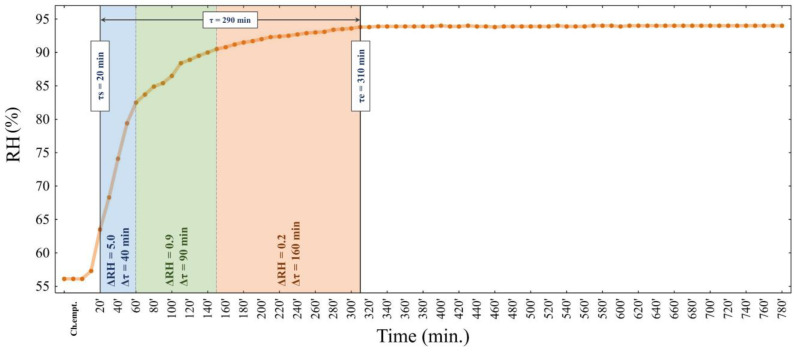
Relative humidity RH (%) of mozzarella cheese stored in WL2A1 covering liquid. Different coloured boxes indicate three areas related to the intensity of transfer. Sample coding as in Table 1.

Analysis of diffusion processes was determined based on the leachate. After 5 h 19 min, water diffusion from the mass of the cheese stored in the covering liquid, which had a ratio of lactose to citric acid of 2:1 (WL2A1), to the outside was only 0.05 mL. These were the first signs of a leachate (Figure 2). The largest total leachate due to diffusion was observed after 17 h from removing the cheese from the liquid and quartering (4.53 mL; Figure 3). Further analysis after this time showed that the diffusion processes from the cheese mass were reduced to zero. At the same time, the last signs of diffusion processes were observed (Δv = 0.06 mL).

Similar studies on the leachate of the water–fat fraction were carried out on cheese made from a mixture of cow’s and sheep’s milk (CSB) in a ratio of 70:30, which was stored in traditional brine [30]. When the leachate results for WL2A1 cheese and CSB cheese were compared, it was found that the leachate for the CSB cheese made from cow’s and sheep’s milk in 70:30 ratio stored in brine was much larger in total. Moreover, it occurred much faster, already within the first hour after unpacking and quartering (0.22 mL). The sum of the leachate was 24.55 mL, which was almost 5.5 times more than for WL2A1 cheese. This proved that storing the cheese in a covering liquid with a modified composition could positively affect reducing the amount of leachate from the mozzarella cheese mass.

The functional properties of mozzarella cheese depend on changing the distribution of water in the cheese, especially during the initial period of storage [31,32,33]. Correia Gonçalves and Cardarelli [34] reported that changes in the water state in cheese occurred during the first 3 weeks of refrigerated storage. In the case of mozzarella cheese, considering the short storage period of this product, this is crucial for consumer satisfaction.

The analysis of water mobility expressed as relative humidity RH (%) at a specific time showed that the stabilisation of the equilibrium relative humidity in the surroundings of the cheese sample taken from WL2A1 covering liquid lasted approx. 5 h (Figure 3). The phase of translational water movement in this cheese lasted 290 min (τe = 310 min). At that time, three areas related to the intensity of transfer were identified. In the first one (for τs = 20 min), the average ΔRH was 5.0 for Δτ = 40 min; in the second, ΔRH = 0.9 for Δτ = 90 min; and in the third, ΔRH = 0.2 for Δτ = 160 min. The last one was the transformation of the translational motion of water molecules before entering the surface exchange region and establishing an equilibrium state. By parameterising the kinetics of translational movement, it was shown that the time to reach the maximum velocity was τm = 20 min for vm = 0.31 RH/min.

### 3.3. Characteristics of Colour and Examination of Changes during Later Stages of the Technological Cycle

The yellowness index (YI) of cheeses stored in double acid covering liquid (WA2) and baked on pizza did not differ from the YI of the control cheese (Table 3). However, for WA1, this value was much higher (YI = 56.68 ± 0.8). Any addition of lactose to the covering liquid resulted in an increase in the YI of the cheeses after baking compared to the control. However, the amount of lactose added did not influence YI, nor was there a difference between the YI of cheeses stored in WA1 and WL2A1 covering liquid (*p* > 0.05).

The highest chrome (C*) for cheese after baking was found for the cheese stored in WL2A1 and WA1 covering liquid. In the case of other cheeses after baking, the composition of the covering liquid did not affect the chrome (*p* > 0.05). After baking, there were no differences between the chrome of the control cheese (W) and all other cheeses stored in covering liquids with modified composition, except for cheese from the WL2A1 covering liquid (*p* > 0.05).

Assessment of changes in direction in the main descriptors regarding the texture, melt and colour showed that for WL1 cheese, there was no change compared to the control (Table 4). The only cheeses that showed improvement (+) for texture were WL2 and WL2A1 cheeses. Improvement of the melt was observed for cheeses stored in WL1A1, WL1A2 and WL2A1 covering liquids. The colour improved for cheeses WL2, WA1, WL2A1 and WL2A2. In general, the greatest improvement was demonstrated for cheeses stored in covering liquid WL2A1 (3+).

**Figure 4:**
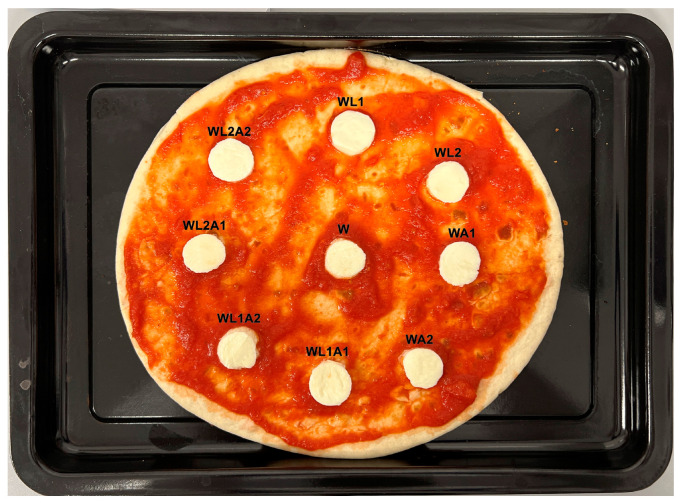
Mozzarella cheese samples before the pizza baking test. Sample coding as in Table 1.

**Figure 5:**
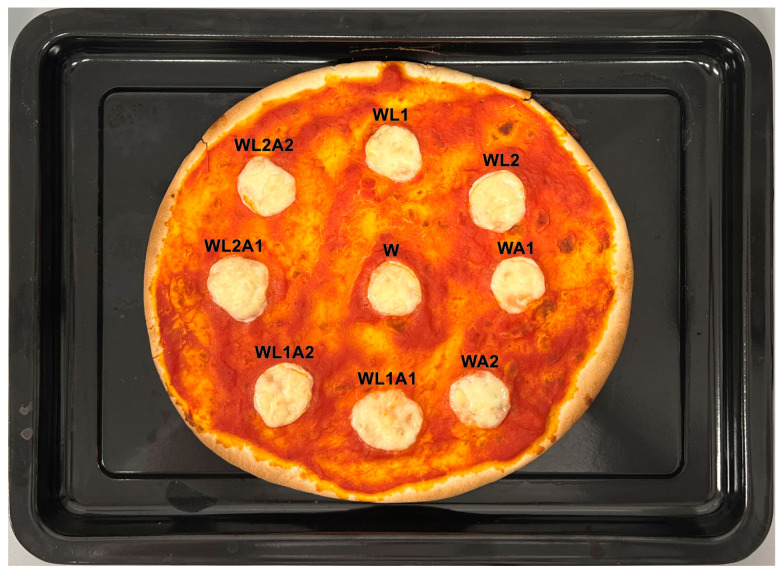
Mozzarella cheese samples after pizza baking test. Sample coding as in Table 1.

The evaluation of the descriptors showed that storing mozzarella cheese in a covering liquid with the addition of lactose and/or citric acid may influence the direction of changes that occur during further use of the cheese, e.g., as a pizza topping (Figure 4 and Figure 5). There was no deterioration of the melt detected for the studied cheeses. It was observed that the addition of only citric acid to the liquid, both in single and double amounts (WA1 and WA2), negatively affected the texture. This was also demonstrated for cheeses stored in liquid with added acid and a single amount of lactose (WL1A1 and WL1A2). The results also indicated a possible protective effect against negative changes after baking for the covering liquid with double the amount of lactose added. The only cheese that showed improvement for all descriptors was WL2A1 cheese.

**Figure 6:**
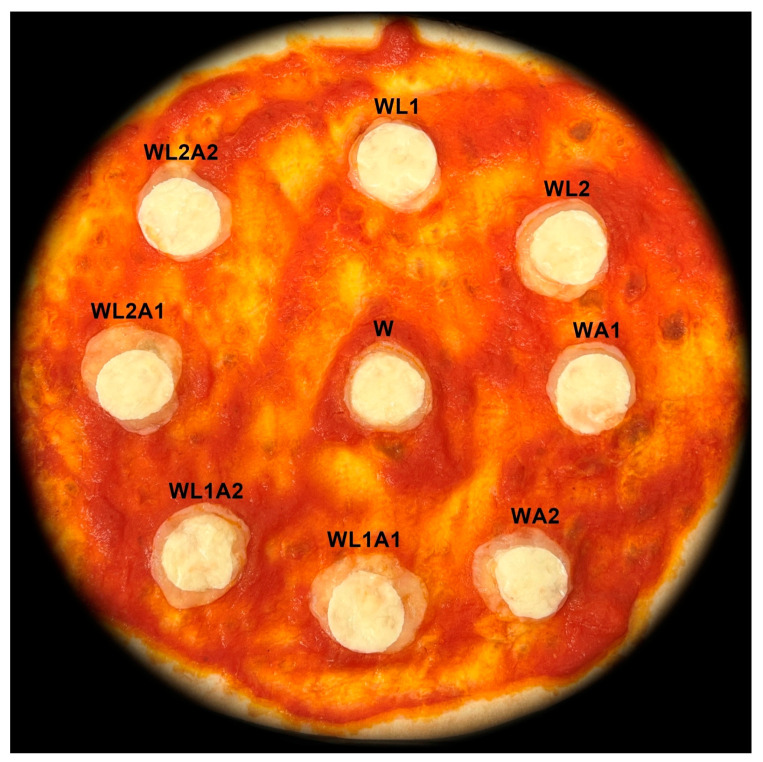
Simulation of mozzarella cheese melting degree during pizza baking test. Sample coding as in Table 1.

The pizza baking test is commonly used to assess changes that occur in different cheeses during their culinary use. For example, Wadhwani et al. [35] used the pizza baking test to assess stretchability in conjunction with the fork test. Sutariya et al. [18] assessed the melting, browning, and stretch characteristics of mozzarella cheese using the pizza baking test. However, scientists raise the possibility of using this test mainly to characterise cheese, among others, with changed characteristics, e.g., reduced fat content. To date, there is no literature data regarding the use of the pizza baking test for cheeses whose characteristics are created using innovative covering liquids used for storage. The changes that occur during baking can also be presented in the form of a melting degree simulation (Figure 6) as a way to easily compare these changes.

## 4. Conclusions

Storing mozzarella cheese in a covering liquid with an altered composition allows for modelling the quality of the fresh cheese and its further culinary use. The observed characteristic changes in the cheeses reflected the parameters of the covering liquids. Storing mozzarella cheese in novel covering liquids with added citric acid and/or lactose significantly affects the mass of the cheese. It was found that changes in cheese mass were the smallest in the case of mozzarella cheese stored in WL2A1 covering liquid (simultaneous addition of lactose and citric acid in the amount of 20 g/kg and 0.42 g/kg, respectively). This cheese was also rated as the best in terms of the characteristics of changes that occur when it is used as a pizza topping.

Storing mozzarella cheese in a covering liquid with added lactose and citric acid 2:1 (WL2A1) significantly influenced water diffusion, which was closely related to the mass of the cheese. Leachate of the water–fat fraction was observed from WL2A1 cheese, which occurred relatively late and in a very limited amount.

The results obtained in this work show that the characteristics of mozzarella cheese, regardless of the way it is consumed, can be created not only by changing the composition of the cheese itself but also by changing the composition of the covering liquid in which it is stored. This allows for new possibilities that can positively influence the consumer’s perception of this product. The fact that the culinary and functional characteristics of mozzarella cheese can be created by modifying the composition of covering liquids also opens up a wide range of scientific possibilities. Modifying the composition of the covering liquids may allow for a completely new look at mozzarella cheese, which should be the basis for further scientific research. Future research should also take into account the aspect related to the packaging sector, which may be interested in creating packaging for covering liquid–stored mozzarella cheeses with resealable systems. This will allow the consumer to store the mozzarella cheese in the covering liquid without having to store it outside the packaging if the cheese is not consumed in its entirety at once.

## Figures and Tables

**Table 1:** The amount of lactose and citric acid added to whey (*w*/*w*) during the preparation of innovative covering liquids. Presentation of sample coding.

Sample Codes *	Lactose ** (g/kg)	Citric Acid (g/kg)
W	–	–
WL1	10.0	–
WL2	20.0	–
WA1	–	0.42
WA2	–	0.84
WL1A1	10.0	0.42
WL1A2	10.0	0.84
WL2A1	20.0	0.42
WL2A2	20.0	0.84

* W—Control sample; ** Applies only to lactose added to whey when preparing the covering liquids.

**Table 2:** Physicochemical properties of the covering liquids with modified composition before (**B**) and after (**A**) storing of cheese.

Sample Codes		a_w_	pH	Freezing Point (°C)	Salinity (g/dm^3^)	Conductivity (mS/cm)
W	B	0.9906 ± 0.001 ^de^	6.52 ± 0.0 ^i^	−0.676 ± 0.00 ^m^	8.703 ± 0.02 ^ij^	35.62 ± 0.3 ^f^
	A	0.9843 ± 0.002 ^abcde^	6.67 ± 0.0 ^k^	−0.695 ± 0.00 ^j^	8.397 ± 0.01 ^h^	34.45 ± 0.1 ^de^
WL1	B	0.9911 ± 0.002 ^e^	6.47 ± 0.0 ^h^	−0.729 ± 0.00 ^g^	8.813 ± 0.03 ^jk^	35.56 ± 0.1 ^f^
	A	0.9858 ± 0.002 ^bcde^	6.68 ± 0.0 ^k^	−0.720 ± 0.00 ^h^	8.383 ± 0.02 ^gh^	33.97 ± 0.3 ^bcd^
WL2	B	0.9915 ± 0.003 ^e^	6.47 ± 0.0 ^h^	−0.785 ± 0.00 ^c^	7.570 ± 0.04 ^b^	33.66 ± 0.2 ^b^
	A	0.9835 ± 0.003 ^abcde^	6.61 ± 0.0 ^j^	−0.772 ± 0.00 ^d^	6.773 ± 0.14 ^a^	32.87 ± 0.0 ^a^
WA1	B	0.9851 ± 0.000 ^abcde^	5.89 ± 0.0 ^d^	−0.682 ± 0.00 ^l^	8.907 ± 0.02 ^k^	35.63 ± 0.2 ^f^
	A	0.9770 ± 0.001 ^a^	6.52 ± 0.0 ^i^	−0.705 ± 0.00 ^i^	8.400 ± 0.03 ^h^	34.34 ± 0.1 ^cde^
WA2	B	0.9815 ± 0.001 ^abc^	5.35 ± 0.0 ^b^	−0.689 ± 0.00 ^k^	8.697 ± 0.02 ^ij^	34.71 ± 0.2 ^e^
	A	0.9785 ± 0.001 ^ab^	6.40 ± 0.0 ^fg^	−0.708 ± 0.00 ^i^	8.430 ± 0.01 ^h^	34.46 ± 0.1 ^de^
WL1A1	B	0.9824 ± 0.001 ^abc^	5.79 ± 0.0 ^c^	−0.738 ± 0.00 ^f^	8.620 ± 0.03 ^i^	34.83 ± 0.1 ^e^
	A	0.9857 ± 0.001 ^bcde^	6.43 ± 0.0 ^gh^	−0.730 ± 0.00 ^g^	7.813 ± 0.07 ^c^	33.81 ± 0.0 ^bc^
WL1A2	B	0.9829 ± 0.001 ^abcd^	5.33 ± 0.0 ^b^	−0.745 ± 0.00 ^e^	8.083 ± 0.02 ^d^	34.46 ± 0.2 ^de^
	A	0.9889 ± 0.001 ^cde^	6.36 ± 0.0 ^ef^	−0.735 ± 0.00 ^f^	8.153 ± 0.05 ^de^	33.78 ± 0.1 ^bc^
WL2A1	B	0.9824 ± 0.001 ^abc^	5.83 ± 0.0 ^c^	−0.793 ± 0.00 ^b^	8.337 ± 0.02 ^fgh^	34.83 ± 0.6 ^e^
	A	0.9842 ± 0.007 ^abcde^	6.40 ± 0.0 ^fg^	−0.771 ± 0.00 ^d^	8.080 ± 0.05 ^d^	33.65 ± 0.0 ^b^
WL2A2	B	0.9850 ± 0.001 ^abcde^	5.12 ± 0.0 ^a^	−0.808 ± 0.00 ^a^	8.253 ± 0.04 ^efg^	34.62 ± 0.3 ^e^
	A	0.9874 ± 0.006 ^cde^	6.34 ± 0.0 ^e^	−0.784 ± 0.00 ^c^	8.230 ± 0.04 ^ef^	33.72 ± 0.0 ^b^

^a–m^ Means within a row with different superscripts differ (*p* < 0.05). Mean ± Standard Deviation. Sample coding as in Table 1.

**Table 3:** Colour parameters of mozzarella cheese stored in covering liquids after baking on pizza.

Sample Codes	L *	WI	YI	C *
W	40.27 ± 0.2 ^bc^	61.38 ± 0.2 ^c^	47.40 ± 0.5 ^d^	14.14 ± 0.1 ^d^
WL1	40.07 ± 0.4 ^bc^	61.80 ± 0.4 ^c^	52.20 ± 0.6 ^f^	15.06 ± 0.1 ^de^
WL2	39.23 ± 0.0 ^b^	62.66 ± 0.0 ^cd^	52.13 ± 0.3 ^f^	15.29 ± 0.0 ^de^
WA1	37.40 ± 0.2 ^a^	64.52 ± 0.2 ^d^	56.68 ± 0.8 ^g^	15.64 ± 0.3 ^de^
WA2	40.58 ± 0.1 ^bc^	61.17 ± 0.1 ^c^	48.56 ± 0.6 ^de^	14.51 ± 0.3 ^de^
WL1A1	40.88 ± 0.3 ^c^	61.09 ± 0.3 ^c^	51.41 ± 0.3 ^ef^	15.37 ± 0.0 ^de^
WL1A2	40.21 ± 0.4 ^bc^	61.70 ± 0.3 ^c^	51.81 ± 0.3 ^f^	15.23 ± 0.1 ^de^
WL2A1	40.34 ± 0.2 ^bc^	61.79 ± 0.2 ^c^	53.78 ± 0.6 ^fg^	16.05 ± 0.1 ^e^
WL2A2	40.63 ± 0.4 ^bc^	61.22 ± 0.4 ^c^	50.82 ± 0.2 ^ef^	14.96 ± 0.1 ^de^

^a–g^ Means within a row with different superscripts differ (*p* < 0.05). Mean ± Standard Deviation. L *–brightness, WI—white index, YI—yellowing index, C *—chrome. Sample coding as in Table 1.

**Table 4:** Direction of changes in the main descriptors of mozzarella cheese stored in covering liquids with a modified composition after baking on pizza.

Descriptor	WL1	WL2	WA1	WA2	WL1A1	WL1A2	WL2A1	WL2A2
Texture	0	+	−	−	−	−	+	0
Melt	0	0	0	0	+	+	+	0
Color	0	+	+	−	−	−	+	+
Σ	0	2+	0	2−	1−	1−	3+	1+

Changes in relation to the control sample W: “0” as no change; “+” as improvement; “−” as deterioration. Sample coding as in Table 1.

## Data Availability

The authors confirm that the data supporting the findings of this study are available within the article.
